# Health care: life stories by trans women in Colombia

**DOI:** 10.1186/s12939-023-01859-w

**Published:** 2024-04-30

**Authors:** Juan Carlos Zapata Mayor, Paula Andrea Hoyos Hernández

**Affiliations:** Pontificia Universidad Javeriana Cali, Cali, Colombia

**Keywords:** Transgender, Health Services for Transgender Persons, Gender identity, Gender equity, Sexual and gender minorities, Colombia

## Abstract

**Background:**

In Colombia, health care for people with trans life experiences is characterised by countless barriers to health services and care. Commonly, trans people have experienced stigma and discrimination among health professionals, a lack of services and professionals specialized to guarantee affirmative processes from non-hegemonic gender perspectives, and there exists a marked pathologization and medicalization of services. Therefore, it is necessary to provide affirmative health services to improve health and well-being from the recognition of their needs and experiences. The article describes life narratives about health care for the gender transitions of trans women in Colombia.

**Methods:**

A qualitative narrative study was conducted with 139 trans women in seven cities in Colombia. In-depth interviews and discussion groups were conducted between June 2019 and March 2020. Data were analyzed using thematic analysis and the Atlas Ti cloud program. National and international ethical guidelines were followed in the development of the research.

**Results:**

This research provided an overview of the health experiences of Colombian trans women. They reported their experiences of pathologizing approaches to transgender healthcare, stigma, discrimination, and barriers to accessing preventive, specialized, and regular healthcare services. For this reason, they opted for self-medicated gender transition processes and self-management of health care. An important aspect to consider within healthcare is that not all women want binary gender transition processes.

**Conclusion:**

Participants felt that in Colombia there is a lack of affirmative health care for transgender women and that there are many limitations to care related to the gender transition processes. This exposes them to more situations that violate their rights and influences their lack of confidence and their search for professional health care. In Colombia, it is important to develop strategies for education, information, and communication, as well as a handbook for health workers on specialized healthcare for trans women.

## Background

In Colombia, health care for people with trans life experiences is characterised by innumerable barriers to health services and care. These include stigma and discrimination among health professionals [1, 2], a lack of institutions, services, specialized professionals [3–5], and care pathways that guarantee affirmative processes from non-hegemonic gender perspectives, and a marked pathologization and medicalization of services [4–6]. These factors result in a scarcity of quality health services, health promotion, and access to existing Integrated Health Care Networks (RIAS) in Colombia within the so-called Comprehensive Health Care Model for the General System of Social Security in Health, which claims to provide “real and effective access to health services ensuring accessibility, comprehensiveness, continuity, timeliness, resolvability and equality” [7, p.5]. The influence lack of service to meet the needs of trans communities, and negatively impacts their health outcomes and well-being [8–9].

In recent years, there has been an increasing debate in Latin America about the healthcare processes of trans communities. This process has stimulated a new field of discussion, research, and work for the social and health sciences. Countries such as Argentina, Chile, and Brazil are making progress in processes of inclusion, care, and specialized training for these issues [10–14].

Trans women’s life experiences have been systematically marked throughout history by stigma and discrimination and worse health indicators compared to other trans identities [3, 15–17]. Even in developed countries, discrimination, lack of scientific knowledge regarding trans health, and political, social, cultural, and religious barriers have become an impediment to the free self-determination of identity, and gender expression, such as, through access to transgender hormone therapy. This situation has a negative impact on the health of communities [4, 5, 18–21]. However, five European countries regulate access to transgender hormone therapy from the age of 18 years (Austria, Croatia, Italy, Lithuania, Portugal), and some of these provide for it from the age of 16 if legal representatives provide an endorsement (Ireland, Malta, Netherlands) [22].

The medicalization of their bodies and sexualities has taken place since the nineteenth century, despite demonstrations against it [23]. Trans identities have been medically classified under diagnostic categories that have required psychiatric medical intervention to eliminate, accompany, or legitimize everything that does not fit into the patriarchal and sexist dichotomy [24].

In Colombia, significant progress has been made regarding transgender activism and governmental and academic institutions. These advances reflect the complexity of the issue given the slow progress made to date, and the absence of a specific care route to respond to the health needs of trans identities [1, 4, 21, 25]. This is especially the case when trans women seek to undergo gender-affirmative processes, either through body modifications, hormonal or surgical treatments, or other support, such as psychological care [11], despite legal guidelines that seek to protect and guarantee their rights. These include the National Decree 762 of May 7, 2018, the Care Guidelines for Health Services that Consider a Differential Gender Approach and Non-Discrimination for LGBTI People, United Nations Population Fund (UNFPA) [2], as well as the establishment of the guarantee of their rights through gender reaffirmation procedures that allow trans persons, to express their identity through the correspondence between the sex assigned at birth and their gender identity. These are not considered aesthetic procedures and all healthcare providers are obliged to guarantee trans people adequate and comprehensive access, following Rulings T-771 and T-552 of 2013. Another important step forward is Ruling T-447 of 2019, which establishes that trans people can change their legal names and the sex given on their ID card and that they do not require any medical documentation to support it.

Although the previously mentioned aspects exist, in Colombia, the reality faced by people when accessing these types of services continues to be problematic [21, 25, 26]. Concrete actions are required to strengthen health processes based on clear and mandatory routes for trans identities, which integrate the voices of the people themselves as active agents and experts in their processes.

Considering the picture described above, this study gives an account of several life stories about the health care of trans women in Colombia. It also considers several perspectives which include; the thought of social medicine and collective health in Latin America with the health of trans women in Colombia, the search for trans women’s development, a guarantee of health rights from the postulates of Jaime Breilh, and the social determination of health as a tool for transformation towards new public health to understand the social factors that are linked to the needs, manifestations, and diseases of trans women in their gender transition processes.

The life stories of transgender women will allow us to recognize aspects that are central to strengthening their health and well-being processes and narrowing the existing health gaps in this community. These life stories are configured through the narratives of transgender women, accounting for the meanings that they have constructed from their daily experiences, their explanations and meanings during their backgrounds, and their expectations for the future [27]. Their narratives will allow the construction of affirmative actions for trans-gender people that promote comprehensive health care for this population [28, 29].

## Methods

A qualitative narrative thematic life history study [30] was conducted with 139 trans women in seven cities in Colombia: Cali, Armenia, Calarcá, Jamundí, Bogotá, Bucaramanga, and Cartagena. The inclusion criteria were (a) people who had been self-identifying as trans women for at least the last two years; (b) over 18 years of age; c), living in one of the cities mentioned for at least six months; d) people who freely agreed to participate in the study by giving verbal and written consent. Exclusion criteria were not considered.

The women participated in the Project TranSER: a program to strengthen of full, satisfying, and healthy sexuality among transgender women in Colombia. TranSER was a participatory action research that was carried out between February 2019 and February 2022.

The collection of information was carried out between June 2019 and March 2020, through in-depth interviews conducted by each person on their own, to deepen their socio-demographic characteristics, aspects of sexuality, family and couple, social, biomedical, nutritional, psychological, and occupational aspects (Hoyos-Hernández et al., 2021). This article explored the socio-demographic characteristics, health needs, and healthcare conditions. The latter category emerged in the interview.

In addition, discussion groups took place to collect information from the group and their general experiences on aspects related to (1) Sexuality: meanings and experiences, corporeality, gender identity, gender expression, sex-affective relationships, and sexual satisfaction. (2) Healthy practices about sexuality: self-care, management of emotional states, sexual assertiveness, HIV/AIDS. (3) Risky sexual practices: consumption of psychoactive substances and alcohol, sexual relations without the use of condoms, sex work, sexual violence/abuse, and emotionality. (4) Addressing the gaps in Colombia related to the health of transgender women (Hoyos-Hernández et al., 2021). The discussion groups contained between seven to twelve people and lasted an average of 120 min. This information was used to analyze the gaps related to transgender health in Colombia.

The categories of analysis emerging from the thematic analysis of the women’s narratives (according to the objectives of this study) are described in the results Sect. [31]. It should be noted that the interviews and discussion groups used audio recordings and were transcribed verbatim, then organized using thematic analysis in the Atlas ti cloud program. Consensual Qualitative Research (CQR) was carried out to achieve a reliable transfer of the women’s experiences according to the context in which they took place [32].

### Ethical considerations

This research incorporated reflexivity, positionality, and transdisciplinarity for the construction of knowledge on the health care of trans women.

## Results

A total of 139 women took part in the study. They identified themselves as transfeminine women, who live in seven cities in Colombia: Armenia, Calarcá, Bucaramanga, Cali, Jamundí, Bogotá, and Cartagena. They were between 18 and 62 years old (with an average of 32 years) and 69% belonged to the lower socioeconomic status 1 and 2 (according to the classification in Colombia, which are low-income areas), 50% are high school graduates, or have had some form of higher education; and 58% work as hairdressers, or sex workers. (See Table 1).

**Table 1:** Distribution of trans women by city [33]

Distribution by city	N	Percentage	Ages (Range)	Occupation	
Calarcá	6	4%	19–32	Sex work, social management, activism, styling	
Cartagena	32	23%	19–39	Law, psychology, sex work, activism, queen preparation	
Bucaramanga	25	18%	18–53	Styling, law, activism, social management	
Cali	23	17%	39–58	Sex work, styling, activism	
Armenia	22	16%	19–37	Social management, sex work, activism, styling	
Bogotá	21	15%	24–58	Styling, social management, sex work, acting, art, and culture	
Jamundí	10	7%	20–53	Sex work, styling	
Total	139	100%			

The categories for the analysis were: Perceived current health status, Stigma, and discrimination during health care, Barriers to health care, self-medicated gender transition processes, non-binary gender transition processes, Self-management of health care, and social determination in health. These categories were created based on the analysis carried out by the research team and the participants, taking into account the convergences and divergences that participants expressed in the interviews and discussion groups.

The names used in this section are pseudonyms and do not correspond to the identity or legal names of the trans women participants.

### Perceived current health status

According to the information collected in the discussion groups and the in-depth interviews, the women expressed different perceived current health conditions. Most of them in their life stories report a negative assessment of their physical and psychosocial health, which has a negative impact on their perception of their quality of life.

[...] In my mental health, I have suffered from depression and when I started the transition, I suffered a lot from anxiety and panic attacks [...] (Bucaramanga, personal interview).

[...] At the moment I feel ruled by hormones, in total depression, sometimes I feel like killing myself and sometimes I feel worse, I’ve told the endocrinologist and he says that these are changes that we must deal with, that women feel the same way when they menstruate [...] (Armenia, personal interview).

[I am going to complete eight months without treatment, because they took me out of the health system, because of the change in my ID card number. I have had relapses, but with I have managed to overcome them because I want to go on, because I am the one who looks after my mother financially [...] (Cali, personal interview).

[...] I feel fine, but a week ago I had a little fever and that was because I tried to inject two hormones in a row, one here and one here, and as they were different, one, with, one, they had different names, they were different hormones, and the next day I woke up with a horrible fever, it lasted the whole day, all night, and the next day too. But thank God I feel fine now, and about four days ago, five days ago, I took the test and thank God it was fine, normal, so many things [...] (Bogotá, personal interview).

Women’s narratives explained the presence of anxiety, depression, and stress as part of their mental health. They also highlighted the health needs for gender transition, illness management, and preventive health to ensure overall well-being.

### Stigma and discrimination during healthcare

The participants reported experiences that show stigma and discrimination against transgender people, against their bodies, identities, and gender expressions, as well as care processes that do not adapt to or value their particular needs. In their accounts that reveal this discrimination, there are frequent aspects related to interaction with them the use of masculine names and pronouns and not those corresponding to their gender identities and expressions, as well as to a pathologizing religious assessment.

[...] from when the lady arrived, she addressed me with masculine pronouns, when I looked at her, I said: I am going to ask you something, I want you to be honest, do I have a mustache, or are my features so masculine so that you do not realize that I am a transgender woman, “I’m sorry”, she said. I said that to her: because I really don’t want it to happen again to another girl like me, if you look at my identity, I am legally a transgender woman, so I want you to be more perceptive and not do it again because it is very uncomfortable [...] (Bucaramanga, discussion group).

[...] and then in general medicine, I was being seen by a female doctor who had been treating me and my family for a long time, I knew her, and she referred me straight away, but the psychologist she sent me to, I guess she didn’t know, she was like a very closed-minded lady, very religious.... she just told me that I was wrong, that I was wrong because God and all this... she just told me, no, and she told me things from the Bible, she brought a Bible, so I ended went into crisis and I told her once that I wanted to commit suicide, and from there I was referred to psychiatry [...] (Bucaramanga, personal interview).

[...] Because I think that rather than offending me, he is trampling on my dignity as a woman. Because even if they see me with long hair, in a dress, in a skirt, they are still going to call me a boy. I don’t understand why people are sometimes closed to learning that. That is something very basic... So, I think people should pay more attention to the appearance part to know what they call you. And if they don’t know what to call you, they should ask. But they should not say things like pigeonholing you into something you are not. It is difficult to assimilate, and you struggle every day with that [...] (Bucaramanga, personal interview).

[...] I had a consultation with a psychiatrist as I was telling you earlier, but that was in a hospital far away and they made me wait a long time to be seen by a lady as soon as she saw that I was a trans girl told me that she wasn’t qualified to take on a case like mine, I felt so strange. Am I so different? [...] (Bucaramanga, personal interview).

Stigma and discrimination against transgender people are common in healthcare settings. This exacerbates the structural violence they face on a daily basis.

### Barriers to healthcare

The women refer to barriers related to access to healthcare, which is made clear by the difficulties they have in obtaining medical appointments, either because of severe difficulties in contacting health services by phone, in person, authorizing medical orders, or because they do not have health insurance. However, some of those in the subsidized scheme say that they feel comfortable with the care they are given and that it is free of charge.

Although most of them are affiliated with the Social Security Health System, there have been problems with the change of their ID cards, transfers to other cities, and changes in affiliation status when they have obtained a job, i.e., this is because they stop stopping belonging to the subsidized scheme and become employees that join the contributory health scheme.

[...] they have explained to me what to do, but I don’t know how well, they send me to such and such a place to make an appointment, that, from the appointment to get the authorization, and with the authorization to get the appointment, oh no, no, I hate all that mess [...] (Jamundí, personal interview).

[If I am honest, I have no social security coverage or pension, so why not? I’ll tell you why, because I am in the Sisben (authors’ note: Colombian national system that identifies potential beneficiaries of social programs and which classifies Colombian citizens according to their living conditions and income) health scheme that they gave me, the state gave it to me and I am not going to leave the Sisben for anything in the world, do you know why? because the treatment, all that they have given me has not cost me a penny, and I am better cared for, they have never said no and they have never told me I can’t have that test, surgery, or reconstruction, that they are going to remove my prosthesis because I dislocated it [...] (Cali, personal interview).

[...] The thing is that the health system is something that is, let’s put it this way, macabre, terrible, you can get an appointment... because, that’s what happened, recently when I was with a friend, we went to the health post, we had passed by there the night before, at 3 am, and at that time there were already people waiting for a ticket to join the line [...] (Calarcá, discussion group).

Women’s narratives reveal a health system that is not prepared for gender-affirmative care.

### Self-medicated gender transition processes

In the narratives, women report that stigma, discrimination, and barriers to care have generated a rejection of the system and health care for many of them. Many of them report a lack of trust in healthcare staff and say they have a greater preference for artisanal healing and gender-affirmative practices. The narratives of some trans women show that they resort to homemade, non-certified, or artisanal methods, carried out by themselves, their peers, or another person with experience in such procedures, but who is not certified or qualified. In this regard, it is also noted that although they opt for these procedures, they feel it is important to receive information and support from professionals trained in these areas.

[...] I am also self-medicating. I go to the pharmacy every eight days and ... I take my hormones in pills, but I have not seen a specialist or an endocrinologist or anything [...] (Armenia-Calarcá, personal interview).

[...] I put bakery oil on my breasts and it’s leaking out, it’s bothering me a lot anyway, I have an appointment scheduled for tomorrow [...] (Bogotá, personal interview).

[...] Unfortunately many trans women always feel affected by seeing that other women are more feminine, or that they have different bodies... they seek spaces or techniques that unfortunately affect their bodies a lot. What I think about this, I think that, hey, you must have enough character, and more than character, responsibility for yourself to know that if I just let myself get carried away by an impulse of envy or social pressure, um, I’m going to hurt myself. Personally, I think that if I am going to have any aesthetic treatment to change myself for the better, it should be done by a proven surgeon, who has a degree, because in the end I really want to and I have many dreams and I am not going to lose them by having a bad procedure [...] (Calarcá, discussion group).

In the narratives, women report the preference for affirmative gender processes through their peers and outside the Colombian health system.

### Non-binary gender transition processes

While the participants identified with a transfeminine identity, some identified with a partial or non-binary gender transition process. In this regard, making the gender transition is through their internal, psychological identity and feelings about who they are and how they want to express their gender, either with medical and hormonal treatment, or only with aspects of physical appearances, such as clothing, hair, and accessories.

[...] I feel good about who I am, and how I dress when I go out, every day I look at myself in the mirror, after deciding to be a trans woman, I feel comfortable, I have never felt strange because I guess that was my goal and when you achieve it, you feel good... you have to feel good about who you are and you have to accept yourself, and since I have accepted myself as I am, then I feel great, as I am [...] (Bucaramanga, personal interview).

[...] I had never taken hormones, I wasn’t interested, I think that each being is different, I never liked it and I have been feminine since I was very young [...] (Bogota, discussion group).

In this category, it is important to consider that identity processes such as transfeminine women do not necessarily lead to binary transit processes. This counterbalances the binary and hegemonic construction of gender.

### Self-management of healthcare

As part of the barriers to health care, some of the women report acting as citizens and subjects of rights, through different mechanisms to demand their rights. Some women who have managed their processes through these mechanisms become a support network to accompany other women going through similar situations.

[…] I help a lot of girls, the ones who do not want to be seen by medical staff, and all that, because I handle a lot of petition rights and tutelage and security work nationally, so that has helped me with this issue and with society itself […] (Jamundí, personal interview).

[...] we have a lot of problems with the health system, which is very important I am involved in a case against my EPS (authors’ note: EPSs are organizations that provide healthcare in Colombia. They are also responsible for the affiliation and registration of members and the collection of their contributions.), their procedures… legal action and tutelage…The judge ruled in my favor and now I am in the process of a sex change at the hospital. As a result of this hormonal process and its effect, I have a thyroid condition, because they were not giving me the hormones properly [...] (Bogotá, discussion group).

These results showed the agency of women in guaranteeing their rights.

### Social determination in health

Historically, the processes of health and illness and the lifestyles of trans women (although they have been dynamic and have shown variations according to cultures and periods in time) have generated and perpetuated social structures characterized by rejection, stigmatization, and pathologization. This has already been demonstrated in the previous categories. For example, the biomedical model, certain health disciplines, and some religions have perpetuated these aspects, as part of hegemonic, cisnormative, and patriarchal models. In their narratives, women participants report different aspects constructed on trans identities. These account for those structures that negatively affect their health and well-being, and are permeated in different areas of their lives, such as family, work, relationships, and education.

[Being a trans woman in Bucaramanga is very difficult, it is one of the most difficult cities because I have always been in Medellin, on the coast, and in Bogota but I feel more vulnerable in my city, in my environment than in other cities, because of the machismo here, it’s a Santander idiosyncrasy that is very deep-rooted. The Santander community is centered only on men and women, you cannot be transgender because they will always say “this is a faggot” and what I hate the most in this life is that they want to call me that [...] (Bucaramanga, personal interview).

[…] At least I did not study, not because I did not have the ability, but because at that time the doors closed to us transsexual women in schools, colleges, and in many public places too, because we were transsexuals, they labeled us as if we were sick in the head, or the machismo of society itself did not allow us to be included in society, so that is what happened to me. That’s when I also fell into prostitution [...] (Bucaramanga, personal interview).

[...] I’m in education and I too feel rejected by the education system, I am only able to talk about certain topics, but other topics I cannot talk about some because they don’t think I am able, so I feel rejected in academia and studying for us is terrible [...] (Cartagena, discussion group).

[...] We are strong, because of all that struggle, in one way or another, because if gender were a disease or a vice as they say, we would not live long, because we endure everything we live through socially, we are very strong because in some way it makes us be women, trans people, it is something we carry within. And many, many of us lose very sacred things because we want to build ourselves as women. After all, that is how we want to build ourselves [...] (Armenia, discussion group).

## Discussion

From the life stories of trans women, their narratives show time and time again that their health needs and expectations are not met or fulfilled in a timely, relevant, quality, and continuous manner [28]. This is consistent with the shortcomings in the implementation of the Colombian health system [34] in the seven cities in the study where the participants live, which diverge from the provisions of the Model of Comprehensive Health Care and the goals established for the fulfillment of the Ten-Year Public Health Plan and the Sustainable Development Goals. This reveals that the state (as the body responsible for governing), is not a guarantor of the fundamental right to health. It is allowing discrimination, inequality, and inequity to occur, which increases the vulnerability of this population, especially for those women who seek to undertake gender transitions.

Most of the women participants report that in healthcare contexts they are aggrieved and segregated. They are exposed to discrimination, by not being called their chosen identity names, this even applies to those who have an ID card that clearly states their identity. They also report having limited access to essential social services and many are not in good physical, social, or psychological health.

These results coincide with other studies that report health care that does provide professional interaction with a gender approach [1, 3–5, 13, 34, 35]. By failing to comprehensively address the needs and particularities of trans women, the state, health providers, and legislators cannot make public health decisions that favor the health and well-being of this population [27], which perpetuates the structural barriers in other contexts, and not only in health institutions.

The lack of application of complex models such as Social Determination by the state does not allow the generation of knowledge and actions of those who provide health services to trans women and, therefore, these tend to be reductionist, biologist, and ahistorical [5].

Social Determination through critical epidemiology is an epistemological proposal that serves to understand the processes of health and illness. It provides a conceptual and instrumental method and recognizes how health and illness are socially determined in trans women [29]. Therefore, Latin American Social Medicine (as seen from the gender perspective), makes it possible to observe the health problems of trans women from an integral and complex perspective. It understands that their health needs can be seen beyond the bases of the determinants proposed by the World Health Organization (WHO). Additionally, it provides a glimpse into the health of the individual, her family nucleus, and the interrelationship with her environment, through critical epidemiology.

Pathologization is part of the social determination of their health conditions. Most of the women’s stories demonstrate that discrimination is a concrete expression of stigmatization and the different expressions of violence influence the health care received by trans women because they are part of these social structures. These are maintained by a series of factors that include limited formal and non-formal education on these issues, the construction of knowledge that is not critical or disciplined, and that is not horizontal or collaborative between academic and non-academic communities. The knowledge that is a cisnormative, reductionist, biologist, medicalized, and colonial and that contains illness-centered social representations, which do not make it possible to address the processes of health, illness, and forms of care from complex models that consider the social and environmental determinants that are historically multidetermined [5, 9].

The implementation of an affirmative healthcare model for trans identities would lead to the recognition and adjustment of services and professional interactions based on practices that would remove pathologization and medicalization. This implies a recognition of the gender spectrum that deconstructs cisnormativity and binary expressions. In this regard, it should be noted that not all trans women seek to carry out processes of gender affirmation and transition that involve body modifications through hormone therapy or surgery. Nor do they seek to conform to social stereotypes regarding feminine identities. Therefore, an effective assessment of health, support, and follow-up should promote gender self-determination and offer evidence-based scientific knowledge that respects both binary and hegemonic expressions of being women, as well as those that are not binary or based on hegemonic gender constructs. Professional interaction that is not conditioned by cisnormative and binary gender worldviews should be integrated into healthcare models [4, 5, 25].

## Conclusion

Trans women have unmet needs and expectations about their health status. The state, as the governing body, is not a guarantor of the fundamental right to health as a pillar for society and historically segregated populations. In addition, there is limited action in terms of education, knowledge generation, and awareness carried out by those who provide health services to trans women. There is also a lack of response from the health sector, which leads many trans women to decide to self-medicate, or not to access care, even though there are risks and implications for their health in the short, medium, and long term.

The actors of the Social Security Health System (SGSSS) need to comprehensively address the health needs of trans women and transform the social representations that generate stigma and discrimination. This would contribute to making public health decisions so that the ‘socio-ecosystem’ of trans women can be established and sustained, and not based on impediments, but on a full life and the effective enjoyment of health.

The processes of health and illness need to be recognized dynamically. This should be based on their variations through time and historical space, in interlocution with collective and individual social determinisms in transdisciplinary and in intercultural construction, according to Jaime Breilh’s postulates.

It is also important to review the discursive processes integrated into the regulations and include in the Comprehensive Health Care Guidelines (RIAS) a chapter dedicated to trans care. There should also be processes to address the pathologization of health workers, eliminate barriers, and ensure the adequacy of services through the implementation of gender clinics and specialized clinics. It is equally important to create strategies for education, information, communication, and a manual of specialized care for trans women for health workers. Social Determination should also be implemented in the construction of knowledge from the social and complex reality of trans women, as a fundamental resource to develop actions in public health that generate a real social transformation [29].

In Colombia, training, and sensitization processes are required from universities and continuing, non-formal, and institutional education programs, as well as affirmative institutional policies for trans and non-hegemonic gender identities and expressions.

Finally, trans women’s voices must be included in health care, research, and education processes to build evidence-based practices on this subject [5].

## Data Availability

In a qualitative study, the data is restricted to the authors to guarantee confidentiality.
